# Regulation of yeast DNA polymerase δ-mediated strand displacement synthesis by 5′-flaps

**DOI:** 10.1093/nar/gkv260

**Published:** 2015-03-26

**Authors:** Katrina N. Koc, Joseph L. Stodola, Peter M. Burgers, Roberto Galletto

**Affiliations:** Department of Biochemistry and Molecular Biophysics, Washington University School of Medicine, Saint Louis, MO 63110, USA

## Abstract

The strand displacement activity of DNA polymerase δ is strongly stimulated by its interaction with proliferating cell nuclear antigen (PCNA). However, inactivation of the 3′–5′ exonuclease activity is sufficient to allow the polymerase to carry out strand displacement even in the absence of PCNA. We have examined *in vitro* the basic biochemical properties that allow Pol δ-exo^−^ to carry out strand displacement synthesis and discovered that it is regulated by the 5′-flaps in the DNA strand to be displaced. Under conditions where Pol δ carries out strand displacement synthesis, the presence of long 5′-flaps or addition in trans of ssDNA suppress this activity. This suggests the presence of a secondary DNA binding site on the enzyme that is responsible for modulation of strand displacement activity. The inhibitory effect of a long 5′-flap can be suppressed by its interaction with single-stranded DNA binding proteins. However, this relief of flap-inhibition does not simply originate from binding of Replication Protein A to the flap and sequestering it. Interaction of Pol δ with PCNA eliminates flap-mediated inhibition of strand displacement synthesis by masking the secondary DNA site on the polymerase. These data suggest that in addition to enhancing the processivity of the polymerase PCNA is an allosteric modulator of other Pol δ activities.

## INTRODUCTION

During lagging strand DNA replication DNA polymerase δ (Pol δ) performs three essential and basic functions in the process. Via DNA-directed DNA synthesis Pol δ catalyzes extension of the short Okazaki fragments generated by DNA Pol α, thereby filling the gap between two successive Okazaki fragments (1–3). During this process Pol δ also proofreads for mis-incorporated bases via its 3′–5′ exonuclease activity, allowing for a relative high fidelity in copying the template strand (4,5). Finally, during Okazaki fragment maturation Pol δ catalyzes strand displacement DNA synthesis through the downstream Okazaki fragment to allow for the generation of 5′-flaps that are substrates for the FEN1 endonuclease (1–3,6,7). Strand displacement by Pol δ and FEN1 cleavage activity must be a highly coordinated process to generate ligatable nicks that are then the substrate of DNA ligase I (nick translation) (8). In this process the amount of strand displacement activity needs to be regulated to avoid generating 5′-flaps that are long enough to bind Replication Protein A (RPA), as RPA binding is inhibitory to FEN1 cleavage (9). In turn, this leads to activation of a secondary pathway for flap processing that involves Dna2 and Pif1 (10,11).

Mutational studies of Pol δ suggest that *in vivo* all of the known functions of the polymerase require its interaction with proliferating cell nuclear antigen (PCNA) (12,13), the homotrimer DNA clamp that encircles dsDNA. It has long been proposed that PCNA functions as a processivity factor for Pol δ (14) as binding of Pol δ to PCNA increases its processivity in DNA synthesis and it stimulates strand displacement activity (2,14). Indeed, in the absence of PCNA *in vitro* DNA Pol δ can extend a primed DNA template but it can only incorporate a very limited number of nucleotides via strand displacement and it cannot complete synthesis through even a short oligonucleotide annealed downstream (2). However, as for other DNA polymerases (15–17) inactivation of the 3′–5′ exonuclease stimulates the strand displacement activity of Pol δ (2), showing that the ability to catalyze strand displacement is an intrinsic property of the polymerase that is otherwise masked in the wild-type enzyme. In other words, the ability of Pol δ to strand displace is counterbalanced by the 3′–5′ exonuclease activity, which degrades the nascent DNA and thereby restores the nick structure (18).

Previous studies have established that PCNA stimulates strand displacement synthesis by Pol δ (2,14). However, in order to determine the intrinsic stand displacement activity of Pol δ at various nick and flap structures, it was necessary to carry out these studies in the absence of PCNA. We have studied the intrinsic strand displacement activity of Pol δ using short model oligonucleotide substrates where different region of the substrate can be easily controlled. We used 3′–5′ exonuclease deficient versions of Pol δ in order to determine the strand displacement synthesis activity of the polymerase without the possibility of subsequent reversal of strand displacement by its exonuclease activity (18). This allowed us to focus on the basic biochemical properties of this enzymatic activity and ask how it is affected by the presence of 5′-flaps of different lengths in the DNA strand to be displaced, and how the presence of the single-stranded DNA binding protein RPA affects the activity, and, finally, how binding to PCNA stimulates strand displacement. This approach allowed us to discover a novel property of Pol δ, which we propose is regulated by PCNA.

## MATERIALS AND METHODS

### Reagents and Buffers

**Figure 1.**
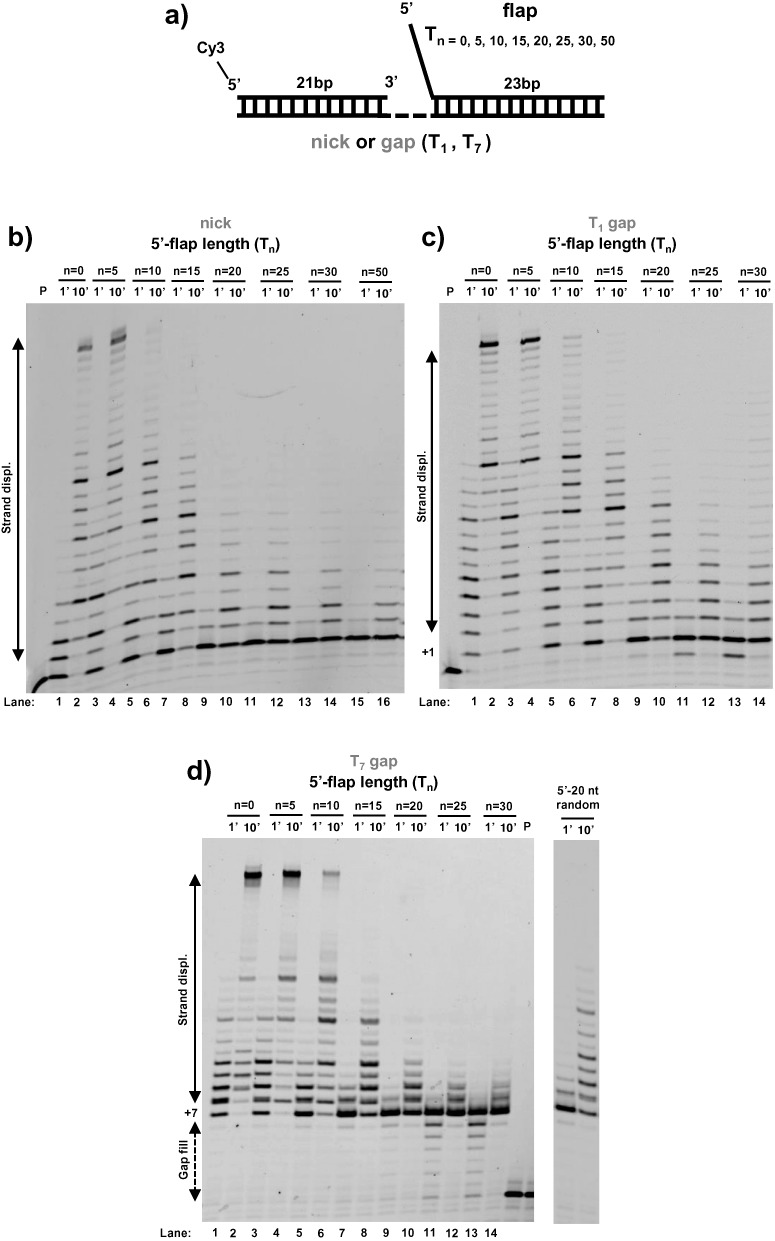
Long 5′-flaps inhibit the strand displacement activity of DNA polymerase δ^DV^. **(a)** Schematic of the DNA substrates used. **(b–d)** DNA primer extension and strand displacement activity of Pol δ^DV^ in Buffer TM (20 mM NaCl, 30°C) as a function of the length (n) of the 5′-flap in the strand to be displaced, for different sizes of the gap in the substrate: (b) nick, (c) T_1_ gap and (d) T_7_ gap. In panel (d) the inhibitory role of a 20 nt 5′-flap of random sequence composition is shown as well.

All chemicals used were reagent grade. All solutions were prepared with distilled and deionized Milli-Q water (18 MΩ at 25°C). Oligonucleotides were purchased from Integrated DNA Technology (IDT, Coralville, IA, USA). The sequences of the oligonucleotides used to build the different substrates (Figure 1a) are as follows:

**P**: 5′-CCGCCGCGGAACTTATTAGTG-3′

with and without Cy3 at 5′-end.

**T1**: 5′-GTGACGGTGTGTGGGTGTGAATC-T_m_-CACTAATAAGTTCCGCGGCGG-3′

with *m* = 0, 1, 7 and biotin at 3′- and 5′-end when indicated.

**T2**: 5′-GTGACACACCCACACACCGAATC-T_m_-CACTAATAAGTTCCGCGGCGG-3′

with *m* = 1 and Cy3 or Cy5 at 5′-end for fluorescence measurements.

**D1**: 5′-T_n_-GATTCACACCCACACACCGTCAC 3′

with *n* = 0, 5, 10, 15, 20, 25, 30, 50 and 5′-end modifications at a given n when indicated.

**D2**: 5′-T_n_-GATTCGGTGTGTGGGTGTGTCAC-3′

with *n* = 0, 25 and Cy3 or Cy5 at 3′-end for fluorescence measurements.

Oligonucleotides with a reverse polarity switch at the last thymidine of the flap were a kind gift from Prof. Lohman (Washington University School of Medicine) and they were synthesized as previously described (19–21). Annealed substrates were prepared by mixing oligonucleotides P, T and D at a ratio of 1 : 1.1 : 1.2 respectively in 10 mM Tris-HCl (pH 8.1), 50 mM NaCl, 5 mM MgCl_2_ and heated at 95°C for 3 min, followed by slow cooling to room temperature.

### Purification of proteins

Wild-type DNA polymerase δ, D520V (Pol δ^DV^), D321A-E323A (Pol δ^01^) were cloned and overexpressed in yeast as previously described (22,23). The proteins were purified with a two-column strategy (22,23) with minor modifications for the wild-type (wt) and exonuclease deficient (DV) proteins. After batch-binding to Glutathione Sepharose 4 Fast Flow GST-affinity resin (GE Healthcare Bio-Sciences, Piscataway, NJ, USA) the GST-tag was removed by overnight on-column digestion with 3C protease in Buffer HEP150 (30 mM HEPES 7.4, 150 mM NaAc, 10% glycerol, 1 mM DTT, 0.01% E10C12, 0.05 mM EDTA, 10 mM NaHSO3, 10 uM Pepstatin A, 10 uM Leupeptin, 0.5 mM PMSF). The cleaved proteins were collected in the flow-through and further purified using a MonoS column. RPA, PCNA and Replication Factor C (RFC) were purified from *Escherichia coli* overproduction strains as described (1,24,25).

### Strand displacement assay

Primer extension and strand displacement reactions were carried out under multiple turnover conditions in Buffer TM (20 mM Tri-HCl pH7.8, 8 mM MgAc_2_, 1 mM DTT, 0.1 mg/mL BSA) with 20 mM NaCl (or otherwise indicated). The experiments were performed by pre-forming a one-to-one DNA and polymerase complex (25 nM final concentration) followed by addition of dNTP mix at a final concentration of 100 μM each (otherwise indicated). For experiments with unlabeled DNAs their concentration is indicated either in the text or figure legend and it is expressed in fold excess relative to the labeled DNA substrate. For experiments with RPA, 50 nM of the protein were either present from the beginning of the reaction or added at the indicated time. For experiments with PCNA a standard loading protocol was followed (2,26). For simplicity the concentrations reported are the final ones after starting the reaction. RFC (25 nM) and PCNA (30 nM) were incubated with a double-biotinylated DNA substrate (25 nM) in the presence of neutravidin (600 nM) and ATP (0.2 mM) for 5 min at 30°C, followed by the addition of Pol δ (25nM) and dNTP mix (100 μM). At the indicate times the reactions were stopped by the addition of 80 mM EDTA, 0.08% SDS. After addition of formamide (50% final), the samples were heated at 95°C for 2 min and analyzed on a 12% denaturing polyacrylamide gel, pre-run for 2 h in 0.5X TBE. The gels were scanned using a Typhoon 9400 Variable Mode Imager (Amersham BioSciences, GE Healthcare Bio Sciences, Piscataway, NJ, USA) and Image J was used for processing and analysis.

### FRET measurements

All the experiments were performed with an L-format PC1 spectrofluorimeter (ISS, Champaign, IL, USA) with temperature controlled by a circulating bath. Cy3 fluorescence time courses were monitored with excitation at 520 nm and emission at 565 nm. The experiments were performed in Buffer TM with 20 mM NaCl at the indicated temperature by pre-forming a complex of 10 nM DNA substrate with 10 nM Pol δ^DV^ followed by the addition of dNTP mix at a final concentration of 100 μM each. For experiments with RPA, 20 nM of the protein was either added at the indicated time or present from the beginning of the reaction. All the experiments were done using a sub-micro cell (Starna, Atacadero, CA, USA) using 130 μl volume and mixing by end. Because of the slow kinetics no loss in amplitude was observed and the data were normalized to the first point after starting the reaction.

## RESULTS

### 5′-flaps longer than ∼10 nt inhibit initiation of strand displacement activity of DNA polymerase δ

Wild-type DNA polymerase δ has primer extension activity but it cannot catalyze strand displacement of a downstream oligonucleotide (Supplementary Figure S1) (2). Therefore, in this work we mainly focused on the *Pol3–5DV* (D520V) form of Pol δ, in which inactivation of the 3′–5′ exonuclease activity has been shown to reveal its capacity for strand displacement (2). For this study the overall structure of the substrates used is shown in Figure 1a. The primer consists of a 21 nt oligonucleotide annealed at the 3′-end of the template and labeled at its 5′-end with Cy3 to allow detection of extension activity by fluorescence. The substrate also contains a 23 nt oligonucleotide annealed at the 5′-end of the template which is separated from the primer region by either a nick, a single (T_1_-gap) or seven thymidines (T_7_-gap). In addition, the 23 nt oligonucleotide to be displaced contains a variable number of nucleotides at its unpaired 5′-end (T_n_ 5′-flap). Consistent with previous observations by Jin *et al*. (2) Pol δ^DV^ catalyzes strand displacement synthesis across a downstream duplex when the strand to be displaced is fully hybridized (no flap) or contains a short 5–10 nt 5′-flap (Figure 1b–d). Surprisingly, however, we found that 5′-flaps longer than 15 nt strongly inhibited the strand displacement activity of Pol δ^DV^, a property not observed previously. Experiments with DNA–Pol δ^DV^ complexes pre-formed in the presence of excess T_1_-gap substrates with different 5′-flaps showed similar results (Supplementary Figure S2). For long 5′-flaps (> 15 nt) the inhibition of strand displacement activity is largely independent of the size of the gap (from a nick to a T_7_-gap, Figure 1b–d) and is also independent of the sequence composition of the flap itself (Figure 1d, T_20_-flap versus 20 nt of random composition). However, as the gap is shortened Pol δ^DV^ becomes more sensitive to the length of the 5′-flap. For example, the T_10_ flap has stronger inhibitory effect on a nick substrate as compared to a T_7_-gap.

Moreover, we note an interesting behavior of the inhibition as function of flap length. During strand displacement a progressively longer 5′-flap is being generated as the primer is being extended. Since the data in Figure 1b–d show that a pre-existing 15 nt 5′-flap is sufficient for inhibition of strand displacement, one would expect that once such a flap has been generated by ongoing strand displacement synthesis, Pol δ^DV^ would stall, leading to partially extended products rather than the full extension observed. Thus, on a DNA substrate containing a pre-existing 10 nt flap, Pol δ should stall after ∼5 nt of strand displacement, leaving a 18 bp stable duplex region. However, this is not observed, as full extension products are produced with this flap containing substrate, when the starting gap is T_1_ or T_7_. This suggests that Pol δ^DV^ senses differently a pre-existing flap as compared to a newly generated one (see Discussion).

**Figure 2.**
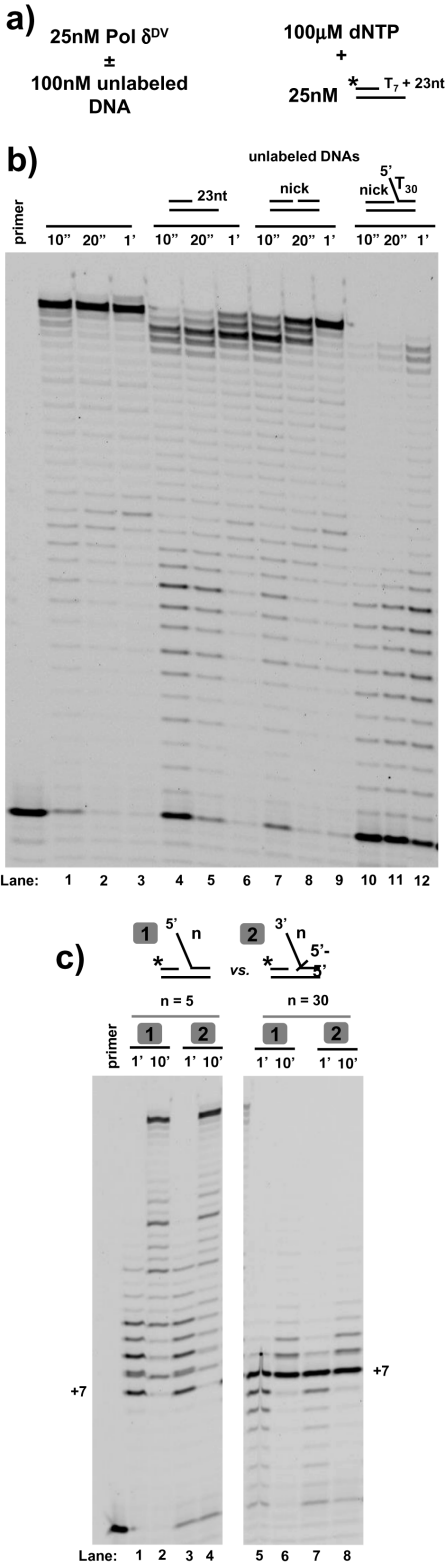
Inhibition of strand displacement is not due to dissociation of the polymerase and is independent of the orientation of the flap. **(a)** Scheme depicting the experiments testing the effect of the 5′-flap on Pol δ^DV^ dissociation from the substrate. **(b)** Primer extension activity in Buffer TM (20 mM NaCl, 30°C) of the reference, labeled primed template by Pol δ^DV^ after incubation in the absence or presence of a 4-folf excess of the indicated unlabeled DNAs. **(c)** Strand displacement activity of Pol δ^DV^ in Buffer TM (20 mM NaCl, 30°C) using a T_7_ gap substrate containing either a 5′-flap (1) or a 3′-flap (2) generated using oligonucleotides with a reverse polarity switch at the end of the flap. The 5 nt flaps are used to control for the effect of the change in polarity itself.

One possible explanation for the observed inhibition is that long 5′-flaps induce dissociation of Pol δ^DV^. In order to test this possibility, we used the experimental strategy in Figure 2a. The assay is based on the rationale that if a long 5′-flap is present at a nick and it induces a low affinity state of the bound Pol δ^DV^ causing more rapid dissociation of the enzyme (compared to complexes formed on substrates without a 5′-flap), a higher primer extension activity should be observed on a reference DNA. Pol δ^DV^ was incubated with a 4-fold excess of unlabeled DNA substrates, followed by the addition of dNTPs together with a labeled template-primer DNA for detection of polymerase activity (Figure 2b). It is evident that the complex of Pol δ^DV^ with a nicked substrate containing a T_30_ 5′-flap is most stable, as it yields the lowest primer extension of the reference DNA. This strongly suggests that the presence of the 5′-flap does not induce dissociation of Pol δ^DV^, but rather stabilizes the enzyme-substrate complex. This is further confirmed by experiments in which the reverse competition was carried out, i.e. Pol δ^DV^ was pre-bound to the reference template-primer, followed by competition by unlabeled nick or flap substrates (Supplementary Figure S3). Moreover, experiments performed as in Figure 2b with a T_1_ gap substrate and different lengths of the flap show that stabilization of Pol δ^DV^ on the substrate occurs at those flap lengths that also show inhibition of strand displacement (Supplementary Figure S4), supporting the suggestion that this inhibition originates from a direct interaction of the flap with the polymerase.

One other simple explanation for the long 5′-flap inhibition of strand displacement activity could be that this activity is associated with an increased K_m_ for dNTPs. However, a 5-fold increase of the concentration of dNTPs did not alleviate the inhibitory effect of the longer 5′-flaps on the strand displacement activity (Supplementary Figure S5).

Because of the length dependence of the 5′-flap inhibitory effect we next tested whether this strictly depends on either the chemical nature or availability of the free 5′-end. For this we used oligonucleotides with the 5′-end of the flap modified with phosphate (P), biotin (bio), digoxigenin (DIG) or a 5′-end bound to neutravidin, streptavidin or anti-DIG antibody (Supplementary Figure S6). With the short (T_5_) control 5′-flap, none of the 5′-end modifications affected the ability of Pol δ^DV^ to carry out strand displacement activity. Analogously, with the longer (T_20_) 5′-flap, none of these modifications was able to relieve the inhibitory effect of the flap, indicating that the observed flap-inhibition of strand displacement activity is independent of the state of the 5′-end of the flap (Supplementary Figure S6).

Next we tested whether inhibition of strand displacement requires a specific orientation of the flap. We used oligonucleotides where a reverse polarity change is introduced at the base of the flap. This allowed us to generate substrates that contain a 3′-end flap rather than a 5′-end one (Figure 2c). With a short (T_5_) control 3′-flap, Pol δ^DV^ could still carry out strand displacement. However, the longer (T_30_) 3′-flap still led to inhibition of strand displacement, showing that regulation of this activity does not require a specific orientation of the flap but rather only the presence of ssDNA.

### Pol δ^DV^ has a secondary DNA binding site that modulates its strand displacement activity

**Figure 3.**
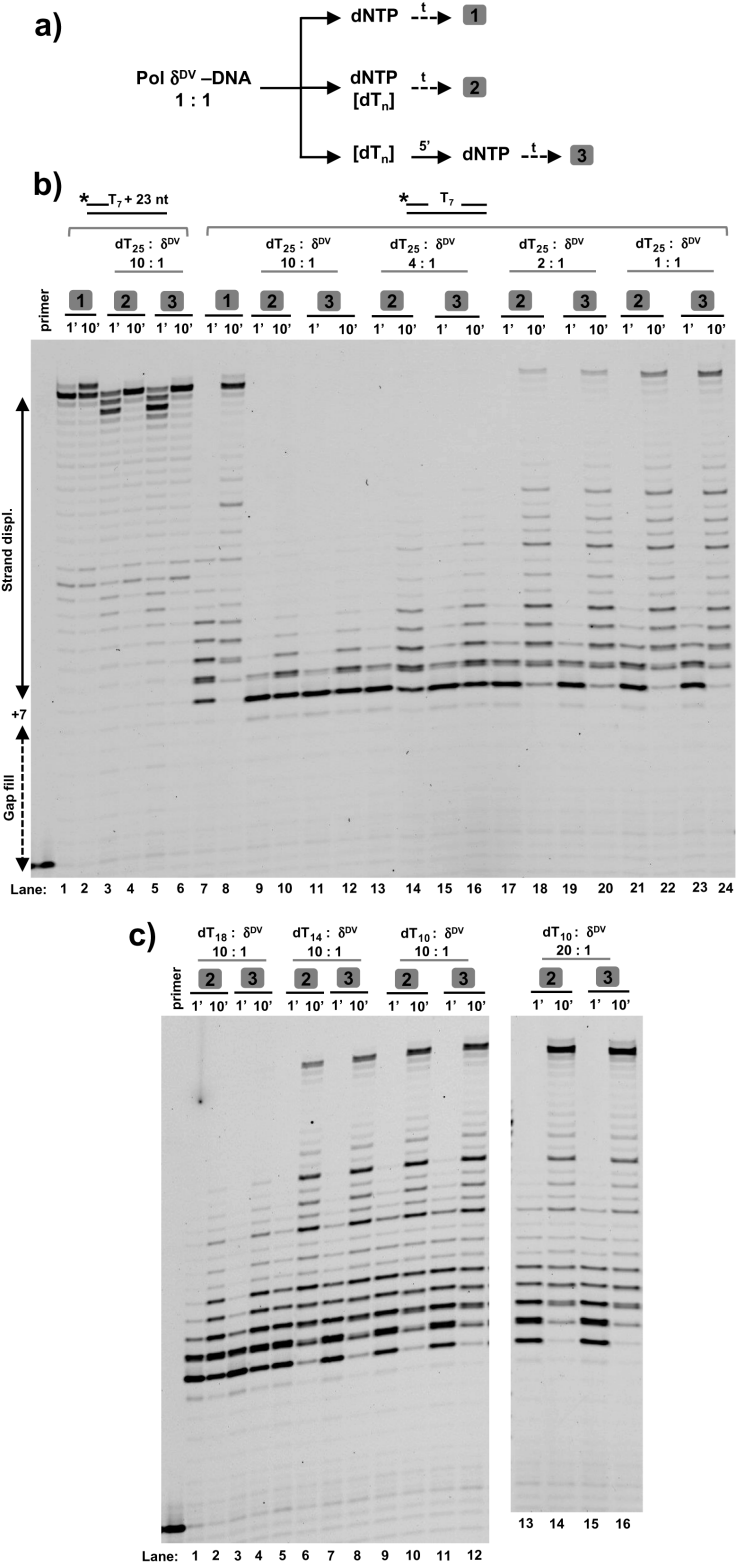
The strand displacement activity of Pol δ^DV^ is inhibited by addition ‘in trans’ of ssDNA. **(a)** Scheme depicting the experimental strategy. After pre-forming a Pol δ^DV^–DNA complex the reactions are started either by (1) adding dNTP, (2) adding dNTP and ssDNA or (3) first adding ssDNA to the pre-formed complex followed by addition of dNTP. **(b)** Effect of different concentrations of dT_25_ on the primer extension and strand displacement activities of Pol δ^DV^ in Buffer TM (20 mM NaCl, 30°C) using either a labeled, primed DNA template or a labeled T_7_ gap substrate without a 5′-flap in the strand to be displaced. **(c)** ssDNA length dependence of the ‘in trans’ inhibition using a 10-fold excess of dT_18_, dT_14_ and dT_10_. For dT_10_ experiments using a 20-fold excess are shown as well. The boxed numbers in (b) and (c) refer to the way the experiments were performed according to (a).

The data presented so far indicate that the observed inhibition of the strand displacement activity of Pol δ^DV^ is a direct consequence of the presence of ssDNA of an optimal length in the 5′-flap. This strongly suggests that direct interaction of the flap with a site on the enzyme leads to inhibition. Also, the observation that for intermediate flap lengths the primer extension activity of Pol δ^DV^ is little affected suggests that the interaction site for the flap must be separate from the primary site responsible for binding of the polymerase to the 3′-end of the primer and the ssDNA template. If a secondary, independent site in Pol δ^DV^ exists and it is accessible, then addition of ssDNA ‘in trans’ may also inhibit the strand displacement activity of Pol δ^DV^. Control experiments in Figure 3a show that addition of a 10-fold excess of dT_25_ together with dNTPs to a Pol δ^DV^-primed DNA complex (or pre-incubation of this complex with excess dT_25_ followed by addition of dNTPs) did not appreciably affect the primer extension activity of Pol δ^DV^. However, the same experiments performed with a T_7_-gap substrate without a 5′-flap show that addition ‘in trans’ of an excess of dT_25_ efficiently inhibited the strand displacement activity of Pol δ^DV^. At least a 4-fold molar excess of ssDNA was needed to elicit strong inhibition, suggesting that the ssDNA present ‘in cis’ as a 5′-flap has a stronger effect than the ssDNA added ‘in trans’. Consistent with the observation that 5′-flaps shorter than 10 nt did not inhibit strand displacement synthesis (Figure 1b–d), we also observed that smaller oligonucleotides added in trans failed to inhibit (Figure 3c). At least a 14-mer was required for partial inhibition, and a 16-mer (not shown) and higher (18 nt) strongly inhibited. Also, the inability of the short dT_10_ to inhibit strand displacement appears to be due to its short length rather than to a weaker affinity, as a 20-fold excess still cannot turn off strand displacement activity (Figure 3b, right). Control experiments in Supplementary Figure S7 showed that the lack of strand displacement activity when ssDNA is provided *in trans* is not due to dissociation of Pol δ from the substrate. Also, although Pol δ has some affinity for ssDNA, dT_25_ is not a good trap for the enzyme and Pol δ can escape the trap on a time scale shorter than the one over which inhibition of strand displacement is observed. These data provide the first experimental evidence that Pol δ^DV^ has a secondary ssDNA binding site that modulates its strand displacement activity.

### RPA relieves the 5′-flap inhibition of strand displacement activity of Pol δ^DV^

**Figure 4.**
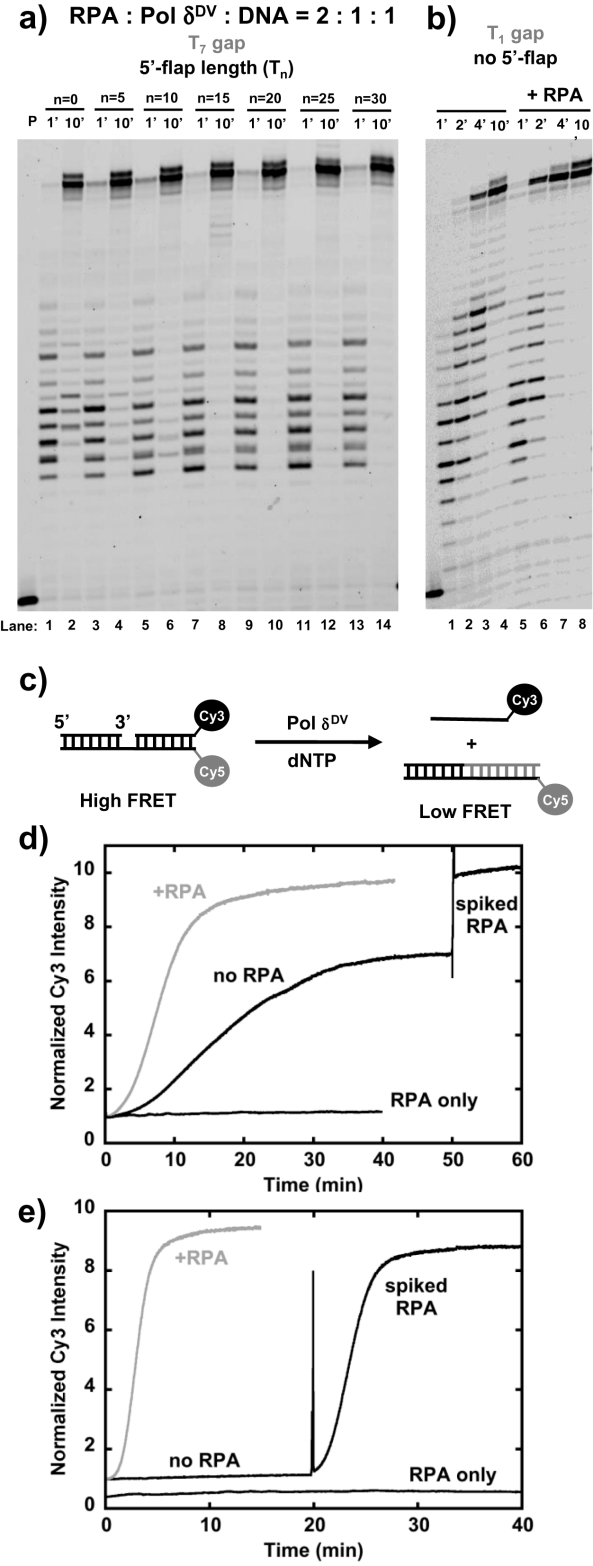
RPA relieves the inhibitory effect of long 5′flaps. **(a)** Effect of RPA on the DNA primer extension and strand displacement activity of Pol δ^DV^ in Buffer TM (20 mM NaCl, 30°C) as a function of the length (n) of the 5′-flap in the strand to be displaced using a T_7_ gap substrate. **(b)** Effect of RPA on a T_1_ gap substrate that does not contain a 5′-flap. **(c)** Scheme depicting the strand displacement assay using the signal from a Cy3–Cy5 couple placed at the end of the duplex region to be displaced. **(d)** Time courses of the Cy3 fluorescence intensity using a T_1_ gap substrate that does not contain a 5′-flap in the strand to be displaced. In black is the time course after addition of dNTP to a pre-formed Pol δ^DV^–DNA complex. In this experiment RPA was added (spiked RPA) 50 min after the reaction had been started. In the gray time course RPA was added from the beginning, before adding dNTP. A control experiment with only RPA added is shown as well. **(e)** Same experiments as in d) but using a T_1_ gap substrate containing a T_25_ 5′-flap in the strand to be displaced. The RPA-only experiment is shown offset for clarity.

Next, we tested whether yeast RPA could relieve the inhibition caused by the long 5′-flap. Figure 4a shows experiments with a T_7_-gap substrate and different lengths of the 5′-flap, performed in the presence of a 2-fold excess of RPA relative to the DNA substrate. RPA completely relieved the inhibition of strand displacement caused by the 5′-flap. The relief of flap-inhibition is not specific to RPA, because *E. coli* SSB can substitute for it (Supplementary Figure S8a). Moreover, relief of flap-inhibition in the presence of RPA was also observed for a flap of opposite orientation (3′-end flap, Supplementary Figure S8b). This would be consistent with the simple idea that binding of RPA to the flap sequesters it, allowing for Pol δ^DV^ to carry out strand displacement. However, we note that as compared to the Pol δ^DV^-only experiments in Figure 1d, for substrates with shorter flaps (e.g. T_5_), full displacement products appeared at the one-minute time point when RPA was present (Figure 4a). This suggests that even for flap lengths that are too short to stably bind RPA (27), the single-strand DNA binding protein stimulates strand displacement. This is further shown in Figure 4b with finer time courses using a T_1_-gap substrate that does not have a 5′-flap (for this substrate oligonucleotides T2 and D2 were used). Again, even though there is no initial binding site for RPA, its presence allowed for full product to be formed at shorter times.

In order to better understand the role RPA plays in stimulating strand displacement, we employed an alternative approach. The assay (shown schematically in Figure 4c) is based on the rationale that if a donor–acceptor couple is placed at the end of the downstream duplex, complete separation of the downstream duplex through strand displacement synthesis by Pol δ^DV^ can be monitored by a change in Forster resonance energy transfer (FRET). For these experiments we used T_1_-gap substrates (as in Figure 4b) with a Cy3 at the 3′-end of the strand to be displaced and a Cy5 at the 5′-end of the template. Addition of dNTPs to a pre-formed complex of Pol δ^DV^ and a DNA substrate without a 5′-flap showed a large change in FRET signal (Supplementary Figure S9a). The black trace in Figure 4d shows a time course for Pol δ^DV^ strand displacement on this substrate, monitoring the change in Cy3 fluorescence intensity after addition of dNTPs. The trace is characterized by an initial lag phase (t_lag_ ∼5 min) followed by a ∼7-fold increase in Cy3 intensity with a t_1/2_ of ∼19 min. Interestingly, The FRET-based strand displacement assay shows slower kinetics than those derived from directly measuring primer extension with the gel-based assay. The reason for this discrepancy is not clear, but one likely explanation is that while the fluorescence assay is only sensitive to release of full product, the gel-based assay, where the reactions are quenched and de-proteinized, monitors formation of full extension product both on and off the enzyme. Nevertheless, both assays show that addition of RPA stimulates the rate of displacement synthesis. In the FRET-based assay with a 2-fold excess of RPA (Figure 4d, gray trace) the time course is characterized by an initial shorter lag of ∼3 min followed by a larger ∼10-fold increase in Cy3 intensity with a shorter t_1/2_ of ∼10 min. The difference in Cy3 signal can be understood when we consider that it has been shown that binding of RPA to ssDNA is accompanied by a fluorescence increase of Cy3 intensity (28). Indeed, addition of RPA at the end of the Pol δ^DV^-only reaction in Figure 4d (black trace) shows an additional increase of the signal, to a similar level to the one observed when RPA is present from the beginning of the reaction. Control experiments monitoring the change in Cy3 fluorescence upon RPA binding to ssDNA further corroborate this point (Supplementary Figure S9b). Moreover, experiments in Supplementary Figure S9c show that the kinetic parameters are little affected when the donor–acceptor couple at the end of duplex was switched around. Also, in this case the maximum signal is independent of the presence in solution of RPA. These data indicate that RPA stimulates strand displacement synthesis even when an initial ssDNA binding site is lacking. This is further supported by experiments performed at lower temperature (Supplementary Figure S10) where the effect of RPA is more pronounced.

Finally, we used the FRET-based assay to examine the effect of RPA on the strand displacement activity of Pol δ^DV^ on a substrate with a T_25_ 5′-flap, which is long enough to provide a strong binding site for the single-stranded DNA binding protein (27). The black trace in Figure 4e shows the time course of Cy3 fluorescence change after addition of dNTPs. In the absence of RPA no signal change was detected for ∼20 min, consistent with the inhibition of strand displacement observed in Figure 1. Addition of RPA after 20 min led to a large signal change with *t*_lag_ ∼ 1.2 min and a *t*_1/2_ of ∼ 3.3 min. Stimulation of Pol δ^DV^ strand displacement activity also occurred when RPA was incubated with the polymerase and the substrate before addition of dNTPs (Figure 4e, gray trace). In this case both the signal change and the kinetics (*t*_lag_ ∼ 1.5 min and *t*_1/2_ ∼ 3 min) were similar to the ones observed when RPA is added after 20 min. Compared to substrates without a 5′-flap (Figure 4d) it is clear that when a site for RPA binding is available (pre-formed) RPA stimulates the reaction. Also, control experiments where only RPA was added to the DNA substrates (Figure 4d and e) showed no change in signal, indicating that even with substrates that contain a long flap, RPA binding itself does not lead to any detectable displacement of the dsDNA.

### Inhibition of strand displacement activity of Pol δ^DV^ at higher NaCl concentrations cannot be relieved by RPA

**Figure 5.**
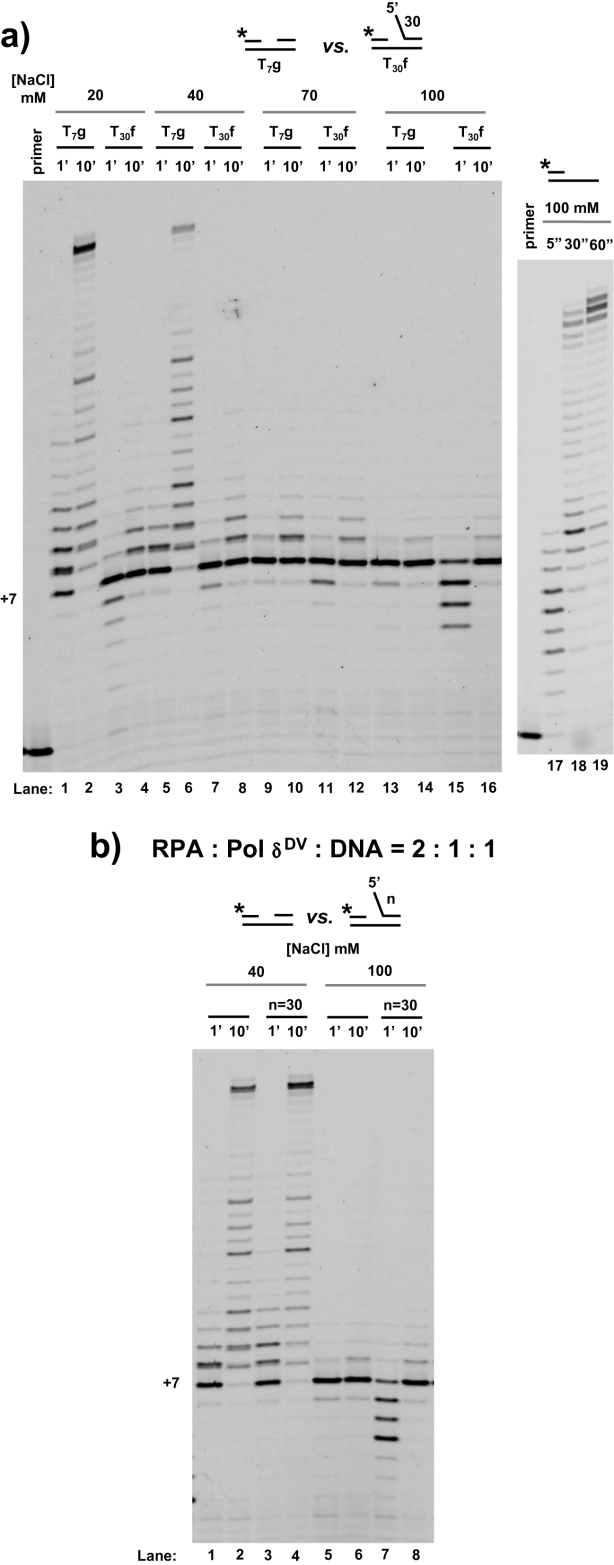
Independent of the presence of a 5′-flap, higher NaCl concentrations inhibit the strand displacement activity of Pol δ^DV^, which cannot be relieved by RPA. **(a)** Strand displacement activity of Pol δ^DV^ in Buffer TM (30°C) in the presence of the indicated NaCl concentration, using a T_7_ gap substrate without or with a T_30_ 5′-flap. The effect of 100 mM NaCl on the extension activity of Pol δ^DV^ using a primed DNA template is shown as well. **(b)** Effect of RPA on the strand displacement activity of Pol δ^DV^ at 40 mM and 100 mM NaCl using the same substrates as in (a).

The data presented so far were collected at low NaCl concentration (20 mM), which is optimal for Pol δ^DV^ activity in the absence of PCNA. Next we examined the effect of increasing NaCl concentration to test the hypothesis that, if the interaction of the ssDNA flap with the secondary site on Pol δ^DV^ were weakened, this could lead to a relief of the inhibitory effect of the flap. Figure 5a shows the strand displacement activity of Pol δ^DV^ at different NaCl concentrations using T_7_-gap substrates either without a 5′-flap or with a T_30_ 5′-flap. Independent of the presence of the flap, the strand displacement activity of Pol δ^DV^ was essentially abolished at NaCl concentrations higher than 70 mM. The lack of strand displacement was not due to an effect of the higher NaCl concentration (70–100 mM) on the primer extension activity of Pol δ^DV^. At these higher NaCl concentrations the polymerase readily filled in the T_7_ gap of the substrate or extended a primed DNA substrate (Figure 5a, right panel). In addition to the regulatory role of the 5′-flap discussed in the previous section, these data show that strand displacement activity is also modulated by salt concentration, as previously observed (2).

Because of the stimulatory effect of RPA on Pol δ^DV^ strand displacement synthesis when a 5′-flap is present (see above), next we tested whether the inhibition observed at the higher NaCl concentrations could be bypassed by binding of RPA to the flap. Figure 5b shows experiments performed at two different NaCl concentrations in the presence of a 2-fold excess of RPA relative to the DNA substrate. At 100 mM NaCl the addition of RPA did not lead to any significant strand displacement DNA synthesis on the substrate with the 5′-flap. The same is true for higher concentrations of RPA (not shown). Binding of RPA to the 5′-flap is not sufficient to relive the inhibition of the activity at the higher salt concentration.

### Binding of Pol δ^DV^ to PCNA relieves all inhibitory effects on strand displacement activity by changing the properties of the polymerase

**Figure 6.**
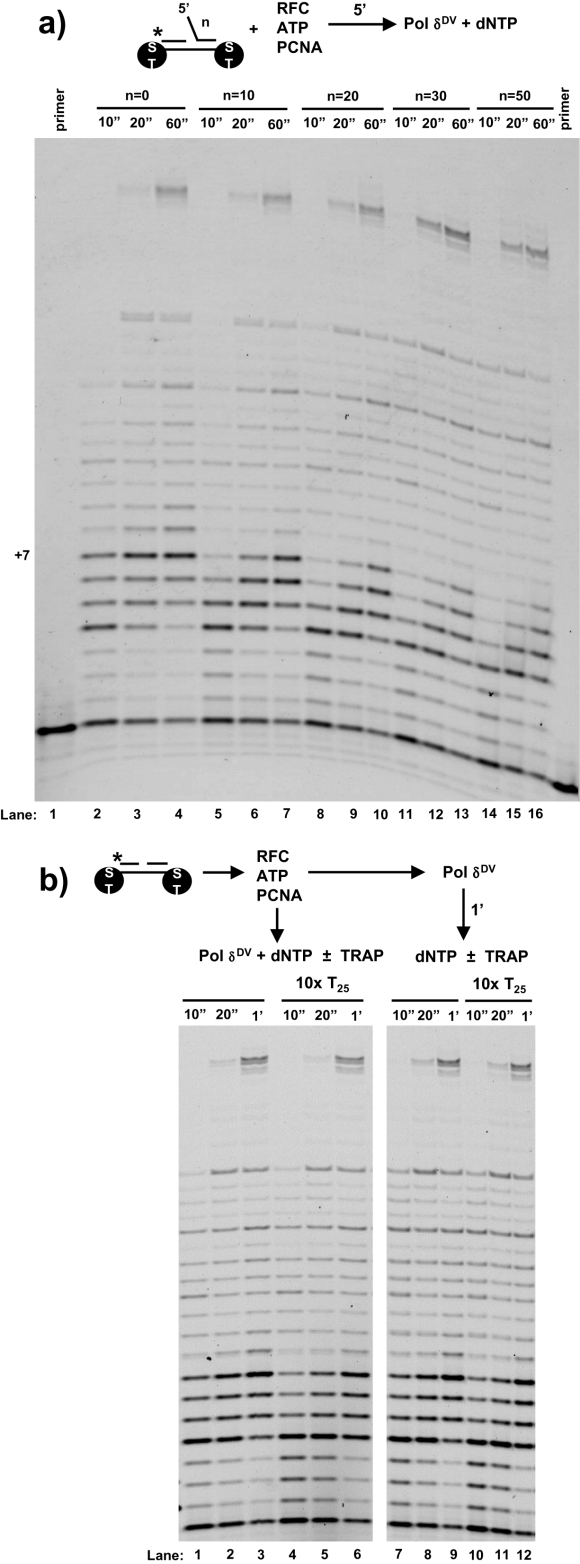
Binding of Pol δ^DV^ to PCNA relieves all inhibitory effects on strand displacement and masks the activity of the secondary site on the polymerase. **(a)** PCNA was loaded on DNA substrates containing streptavidin bumpers using RFC and ATP. The reactions were started by adding Pol δ^DV^ with dNTP. For these experiments T_7_ gap substrates with the indicated length of a 5′-flap were used. **(b)** After loading of PCNA onto a T_7_ gap substrate that does not contain a 5′-flap, the reactions where started either by addition of a solution of Pol δ^DV^ and dNTP with or without a 10-fold excess of dT_25_ or by first adding Pol δ^DV^ for 1 min followed by the addition of dNTP with or without a 10-fold excess of dT_25_.

In the previous sections we examined the intrinsic ability of Pol δ^DV^ to catalyze strand displacement synthesis. However, it has been shown that interaction of Pol δ with PCNA stimulates both its processivity and strand displacement activity (2,3). If the role of PCNA were limited to a function as processivity factor, then the inhibitory effect of the 5′-flap on Pol δ-mediated strand displacement should be independent of its interaction with PCNA. Figure 6a shows assays with T_7_-gap substrates with different lengths of the 5′-flap. For these substrates the 5′- and 3′-ends of the template were modified with biotin and the experiments performed in excess streptavidin to generate ‘bumpers’ that prevent dissociation of PCNA from the substrate (1,29). PCNA was loaded onto the substrate in the presence of RFC and ATP and the reaction started by the addition of polymerase and dNTPs. The data in Figure 6a show that independent of the presence of the flap and its length, PCNA-bound Pol δ^DV^ catalyzed strand displacement synthesis. Thus, binding of the polymerase to PCNA relieves the 5′-flap inhibition observed for the Pol δ^DV^ reactions (Figure 1). Moreover, these experiments were performed at 100 mM NaCl, where Pol δ^DV^ alone cannot catalyze strand displacement on substrates with or without a 5′-flap. Binding of Pol δ^DV^ to PCNA also relieves the higher salt inhibition observed in Figure 5.

It is possible that when bound to PCNA, Pol δ^DV^ is in a conformation that does not allow the 5′-flap to interact with the secondary site on the enzyme and modulate the strand displacement activity. If this were the case the secondary site should still be available and therefore it should be possible to suppress strand displacement activity by adding ssDNA ‘in trans’ as for the experiments in Figure 3. We tested this possibility in Figure 6b using a DNA substrate that does not contain a 5′-flap. The scheme in Figure 6b shows two different ways the experiments were performed. In one case, after PCNA loading Pol δ^DV^ was allowed to bind for 1 min, at which point the reaction was started by addition of dNTPs in the presence or absence of a 10-fold excess of dT_25_ (Figure 6b, right). At difference with what was observed in Figure 3, the addition of dT_25_ did not suppress strand displacement. However, it is possible that binding of Pol δ^DV^ to PCNA occluded the secondary site preventing ssDNA binding. Therefore, the experiments were also performed by starting the reaction with dNTP and Pol δ^DV^ pre-incubated with the dT_25_ (Figure 6b, left). However, the presence of dT_25_ again had no effect on the strand displacement activity of Pol δ^DV^. These data suggest that upon binding to PCNA the secondary ssDNA site of Pol δ can no longer modulate strand displacement activity.

## DISCUSSION

The strand displacement activity coupled to DNA synthesis in the absence or presence of processivity factors, interacting proteins and single-stranded DNA binding proteins, has been studied for several DNA polymerases both on model oligonucleotide and plasmid-sized substrates, and the range of this activity is rather broad. For example, the DNA polymerase from phage ϕ29 is highly active for strand displacement in the absence of any accessory protein (16). The high processivity and strand displacement activity of this enzyme have been shown to depend on the TRP2 domain in the exonuclease that encircles the DNA template, with wedging of this domain into the downstream duplex proposed to allow for strand separation (30,31). Other examples of DNA polymerases that can catalyze strand displacement activity in the absence of accessory factors are the *E. coli* DNA polymerase I and its Klenow fragment (KF) derivative (32), Bst polymerase from *Bacillus Stearothermophilus* (33,34), HIV-1 reverse transcriptase (35–37) and Mip1 polymerase (38). Interestingly for *E. coli* Pol I it has been shown that short 5′-flaps stimulate strand displacement activity (32). The high processivity and strand displacement activity of some of these polymerases (ϕ29, KF, Bst) forms the basis for isothermal strand displacement amplification techniques (39). At the other end of the spectrum of this activity, the DNA polymerases for bacteriophages T4 and T7 have been shown to have low strand displacement activity even when bound to their accessory factors (15–17). For T7 DNA polymerase the enzyme is only able to extend few nucleotides into the downstream duplex (40). For these polymerases it has recently been shown by single molecule magnetic tweezer techniques that their strand displacement activity can be activated by an assisting force that destabilizes the duplex DNA (41). This has led to the proposal that the fork in front of the enzyme exerts a ‘regression pressure’ that inhibits strand displacement. T4 DNA polymerase and DNA polymerase δ both are replicative B-family DNA polymerases, and the ability to carry out strand displacement synthesis is essential for proper Okazaki fragment maturation. Both enzymes carry out increased strand displacement synthesis when their exonuclease is inactivated (2,42).

In this work we showed that the strand displacement synthesis activity of exonuclease-deficient DNA polymerase δ is inhibited by the presence of a long flap at the 5′-end of the DNA strand to be displaced. The inhibitory effect of a 5′-flap, or by long ssDNA added in trans, is not only observed with Pol δ^DV^ (D520V) but also with the exonuclease-deficient Pol δ^01^ (D321A, E323A), showing that this is an intrinsic property of the exonuclease deficient enzyme (Supplementary Figure S11). The inhibitory effect of the 5′-flap does not originate from either a flap-induced dissociation or a large change in the K_m_ for dNTP when in strand displacement ‘mode’. Rather, the ability of strand displacement to be suppressed ‘in trans’ directly points to the presence of a secondary DNA binding site on the polymerase that can modulate this activity. Moreover, the observation that blocking the 5′-end of the flap with a protein does not relieve strand displacement inhibition (Supplementary Figure S4) also suggests that the ssDNA is not threaded into the polymerase to elicit inactivation. This argues that inhibition of strand displacement occurs though interaction of the ssDNA region of the flap with an open site on the polymerase. This is further confirmed by the observation that a flap of opposite directionality (3′-flap) is still inhibitory for strand displacement, showing that it is sufficient for ssDNA to be present (Figure 2c). In addition, the flap-length dependence of the inhibition also shows that Pol δ senses a pre-existing flap differently from a flap that is newly generated during strand displacement. This suggests that the 5′-flap has a stronger effect at the initiation phase of strand displacement; once Pol δ engages (elongation phase), it becomes less sensitive to the flap that is being generated.

The question then is where the secondary DNA site is located within the Pol δ hetero-trimer. The Pol31 subunit links the catalytic Pol3 subunit to the Pol32 subunit, and it is essential for Pol δ function in *Saccharomyces cerevisiae* and in *Schizosaccharomyces pombe* (43,44). No DNA binding sites have been detected in Pol31 or Pol32, or the Pol31–Pol32 complex by electrophoretic mobility shift assays (P. M. Burgers *et al*., unpublished observations). Interestingly, the crystal structure of the human Pol31–Pol32 complex identifies structural similarities to oligonucleotide-binding and phosphodiesterase domains in Pol31 and a winged helix-turn-helix domain in Pol32 (45), all of which could potentially bind ssDNA. However, the possibility also exists that the Pol3 catalytic subunit is a candidate for the presence of the secondary DNA site. The observation that 5′-flaps long enough to inhibit strand displacements activity do not dramatically affect Pol δ primer extension (gap filling) shows that the primary site, comprising the active site and the region for template binding, is separate from the secondary DNA site. Two possible regions in the Pol3 subunit that could contain the secondary DNA site are the exonuclease domain and the N-terminal domain (NTD). The exonuclease domain by definition must bind DNA to perform its function. Intriguingly, for DNA polymerase δ from loach it has been shown that the presence of a short unpaired ssDNA region at the 3′-end of the primer increases the affinity of the polymerase for the substrate (46). The stabilizing effect of the unpaired 3′-flap on the primer has been proposed to originate from interaction with the exonuclease domain, providing further support for the ability of this domain to bind ssDNA. Also, the crystal structure of Pol3 shows that the NTD contains three motifs, two of which have been proposed to bind nucleic acid (47). The most N-terminal motif I resembles DNA binding domains found in ssDNA binding proteins and it is located in the proximity of the 5′-end of the template (47). Its function has been proposed to bind the template ssDNA ahead of the active site (47).

We also showed that the inhibitory effect of a 5′-flap can be bypassed by the presence of a single-stranded DNA binding protein (RPA or SSB). Interestingly, during strand displacement of a mini-circle DNA the presence of RPA appears to limit the size of displaced fragments to ∼ 30 nt (3). The data in this work show, however, that when a pre-formed flap is provided for RPA to bind, the strand displacement activity of Pol δ is stimulated. It is possible that this discrepancy originates from a different way in which RPA can act at the initiation stage versus the elongation phase of strand displacement. The data suggest that it is sufficient for RPA to bind to the 5′-flap to relieve inhibition and the non-specific effect of the single-stranded DNA binding proteins would suggest that the role of RPA is simply to sequester the flap. However, if the only function of RPA were to sequester the flap then in the presence of RPA the strand displacement activity of Pol δ should be the same on DNAs with or without flap. But this is not the case. The FRET-based assays clearly show that in presence of RPA and a 5′-flap long enough to provide a binding site for RPA, Pol δ has better strand displacement activity than on a substrate without a flap (Figure 4). Binding of RPA to the flap facilitates strand displacement. This could be due to a direct interaction of RPA with the polymerase, however no such interaction has been demonstrated to date. Alternatively, human RPA has been shown to diffuse along ssDNA and also transiently open short duplex DNA hairpins, preferentially in the 5′–3′ direction (same as the flap in our substrates) (28). If we were to assume that yeast and human RPA diffuse on ssDNA in the same way and that Pol δ bound to the substrate does not hamper RPA diffusion into the duplex, it is intriguing to speculate that the ability of RPA to melt short duplex DNA could at least in part contribute to the stimulation of strand displacement. It remains to be determined whether under the same experimental conditions the kinetics of Pol δ polymerization and RPA diffusion and dsDNA opening would be compatible for such a mechanism to be in place. At the same time, the inability of RPA to relieve inhibition of strand displacement at higher NaCl concentrations suggests that the mechanism is more complex than transient opening of the dsDNA to allow for Pol δ synthesis. This is further confirmed by the observation that independent of the assay used (gel or FRET) RPA appears to stimulate the strand displacement activity of Pol δ even on DNA substrates that do not contain a RPA binding site to start with.

In addition to the inhibitory effect of the 5′-flap on strand displacement we also showed that, independent of the presence of a flap, NaCl concentrations above 70 mM are sufficient to completely shut down this activity while little affecting primer extension. Moreover, at the higher salt concentration binding of RPA to the 5′-flap is not sufficient to restore strand displacement activity. At physiological salt concentrations Pol δ must interact with an accessory factor in order to catalyze strand displacement. Indeed, binding of Pol δ to PCNA has clearly been shown to stimulate strand displacement activity and all known Pol δ functions *in vivo* appear to be PCNA dependent (12,13,48). Experiments in Figure 6 further reinforce this point by showing that at 100 mM NaCl the polymerase alone is inactive for strand displacement, but the interaction of Pol δ with PCNA restores strand displacement activity. We also showed that once bound to PCNA Pol δ is no longer inhibited by the presence of a 5′-flap. Also, when bound to PCNA the strand displacement activity of Pol δ can no longer be inhibited ‘in trans’, strongly suggesting that the interaction with PCNA has altered the secondary DNA binding site on the polymerase, rendering it unresponsive for regulation of strand displacement. These data show that the interaction of Pol δ with PCNA changes the intrinsic biochemical properties of the enzyme. PCNA is not just an interacting protein scaffold used to increase processivity but may also function as a modulator of polymerase activities. The question remains why Pol δ would contain a secondary site that modulates *in vitro* its strand displacement activity only when the polymerase is not bound to PCNA. The current view of Pol δ function *in vivo* is that it requires its interaction with PCNA; indeed, mutational studies of Pol δ showed that this interaction is essential for cell viability (12,13,48). However, it is intriguing to speculate that some non-essential activities of the polymerase might not be strictly PCNA dependent and in this case the inhibitory role of long ssDNA flaps might be a means to limit strand displacement activity. Alternatively, it could be argued that the ability of PCNA interaction to mask the presence of a secondary, inhibitory site on the polymerase might be a means to channel all Pol δ functions trough PCNA. Finally, while our data show that the secondary site on the polymerase does not significantly affect *in vitro* the strand displacement activity of PCNA-bound Pol δ, this does not exclude the possibility that the site is still accessible and used for some other function.

## SUPPLEMENTARY DATA

Supplementary Data are available at NAR Online.

SUPPLEMENTARY DATA
