# Supplementary material for: Regulation of yeast DNA polymerase δ-mediated strand displacement synthesis by 5′-flaps
Source: Nucleic Acids Res. 2015 Mar 26;43(8):4179–90. doi: 10.1093/nar/gkv260 (PMC4417170; doi:10.1093/nar/gkv260)
Supplement: SUPPLEMENTARY DATA [file supp_43_8_4179__index.html]

Regulation of yeast DNA polymerase δ-mediated strand displacement synthesis by 5′-flaps — Regulation of yeast DNA polymerase δ-mediated strand displacement synthesis by 5′-flaps — SUPPLEMENTARY DATA 

# Regulation of yeast DNA polymerase δ-mediated strand displacement synthesis by 5′-flaps

## SUPPLEMENTARY DATA

**Files in this Data Supplement:**

- SUPPLEMENTARY DATA
