# Supplementary material for: Regulation of yeast DNA polymerase δ-mediated strand displacement synthesis by 5′-flaps
Source: Nucleic Acids Res. 2015 Mar 26;43(8):4179–90. doi: 10.1093/nar/gkv260 (PMC4417170; doi:10.1093/nar/gkv260)
Supplement: SUPPLEMENTARY DATA [file supp_gkv260_nar-00474-v-2015-File008.pdf]

Supplementary Material

Regulation of DNA polymerase δ-mediated strand displacement synthesis by 5'-flaps

Katrina N. Koc, Joseph L. Stodola, Peter M. Burgers and Roberto Galletto

DNA polymerase δ does not catalyze efficient strand displacement.

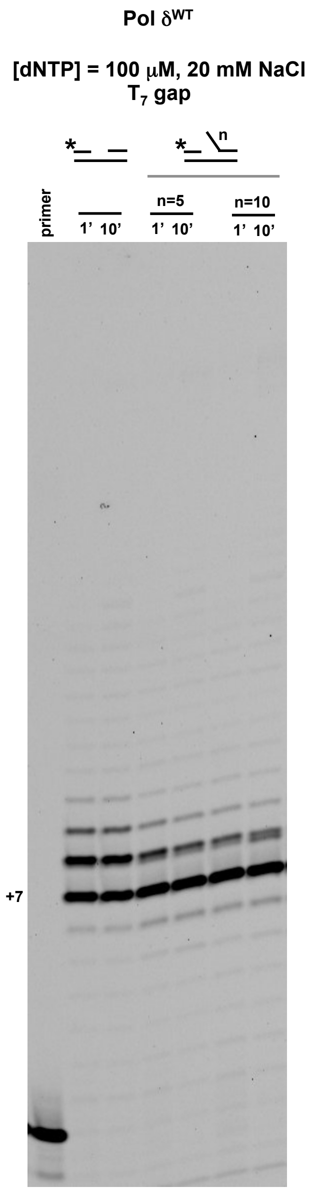

Figure S1. DNA primer extension and strand displacement activity of Pol δ in Buffer TM (20mM NaCl, 30 °C) as function of three lengths of the 5'-flap in the strand to be displaced, using a T_7 gap substrate.

Pol δ^{DV} strand displacement in presence of excess DNA substrates

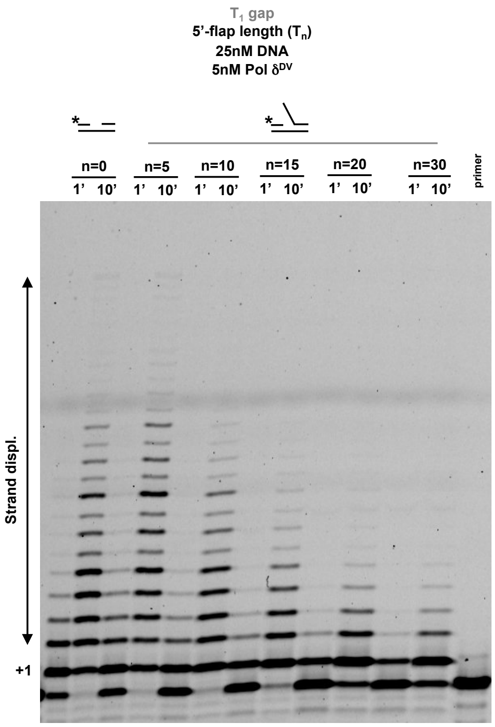

Figure S2. Experiments performed in Buffer TM (20mM NaCl, 30 °C) with five times lower Pol δ^{DV} concentration compared to T_1-gap substrates with different lengths of the 5'-flap.

Direct competition experiments of Pol δ^{DV} from a primed DNA template with unlabeled DNA substrates shows that the polymerase-DNA complex is stabilized by the presence of a 5'-flap.

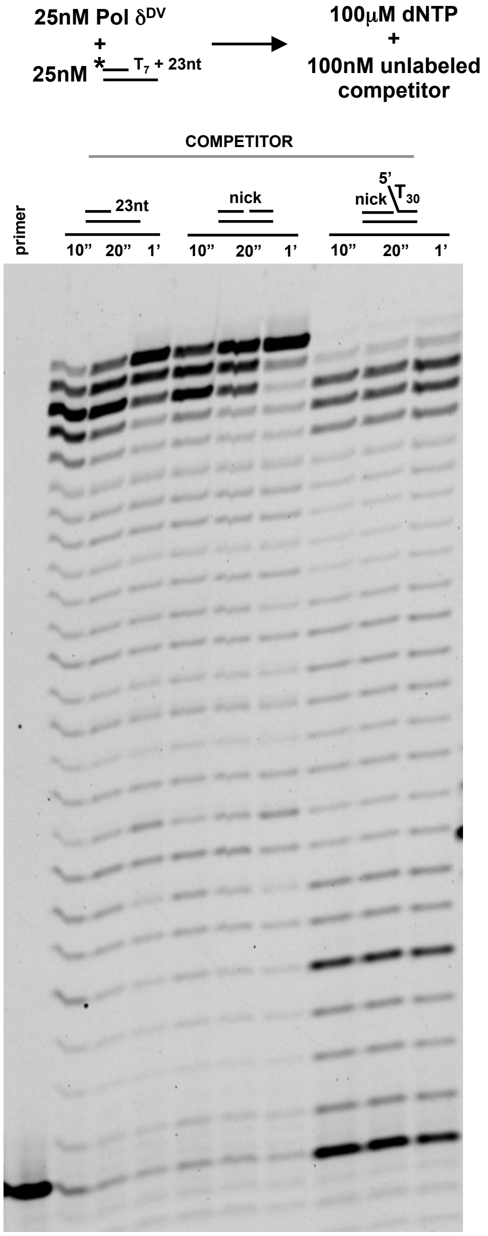

Figure S3. A pre-formed complex of Pol δ^{DV} with a labeled, primed DNA template was mixed in Buffer TM (20mM NaCl, 30 °C) with dNTP and a 4-fold excess of the indicated unlabeled DNA competitor.

With a T_1 gap substrate and different lengths of the flap, stabilization of Pol δ^{DV} on the substrate occurs at those flap lengths that also show inhibition of strand displacement

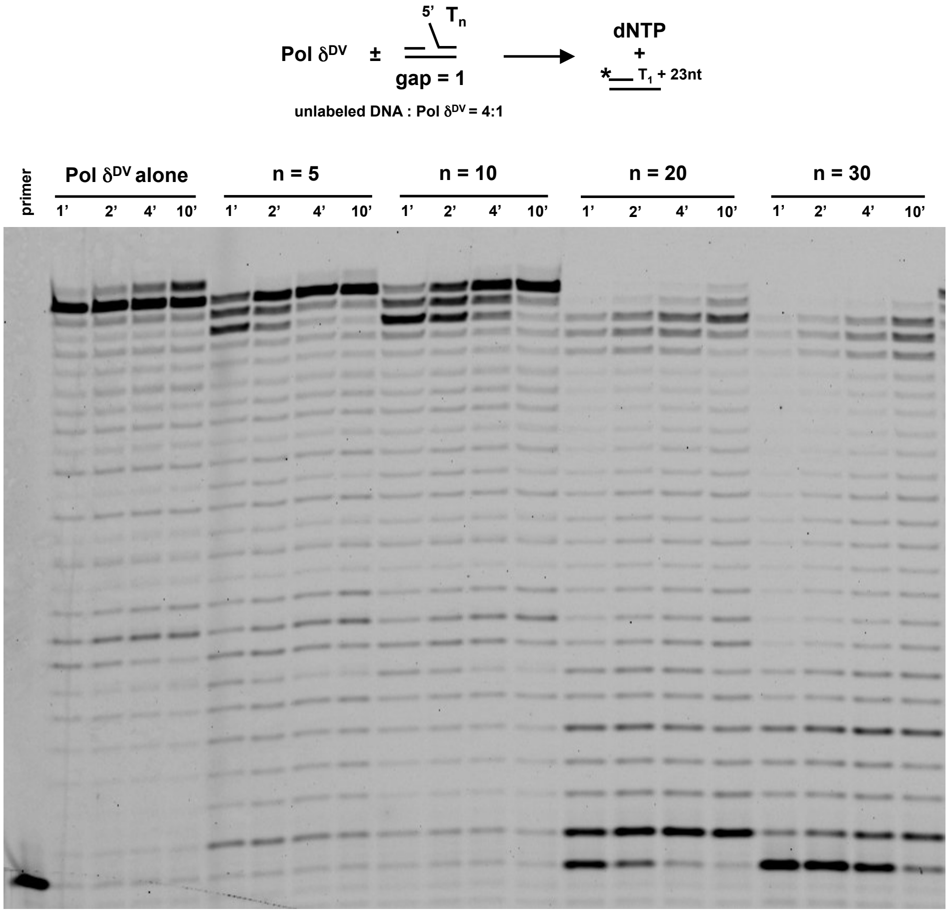

Figure S4. Primer extension activity in Buffer TM (20mM NaCl) at 20 °C of a reference, labeled primed template by Pol δ^{DV} after incubation in absence or presence of a 4-fold excess of the indicated unlabeled DNAs containing a T_1-gap.

The strand displacement activity of Pol δ^{DV} is still inhibited at higher dNTP concentrations

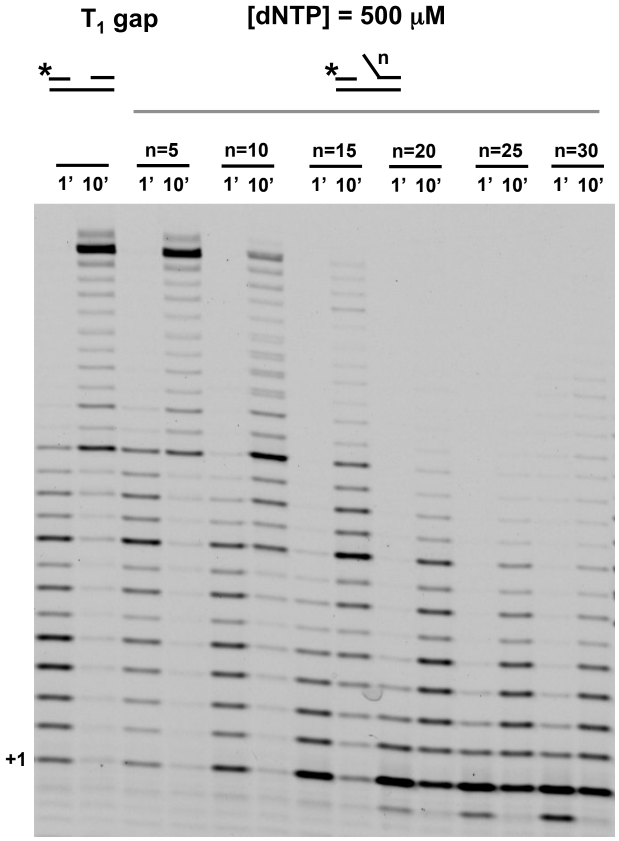

Figure S5. Strand displacement activity of Pol δ^{DV} at 500 μM dNTP in Buffer TM (20mM NaCl, 30 °C) as function of the length (n) of the 5'-flap in the strand to be displaced, using a T_1 gap substrate.

Modification or blocking of the 5'-end of the flap does not affect its ability to inhibit strand displacement.

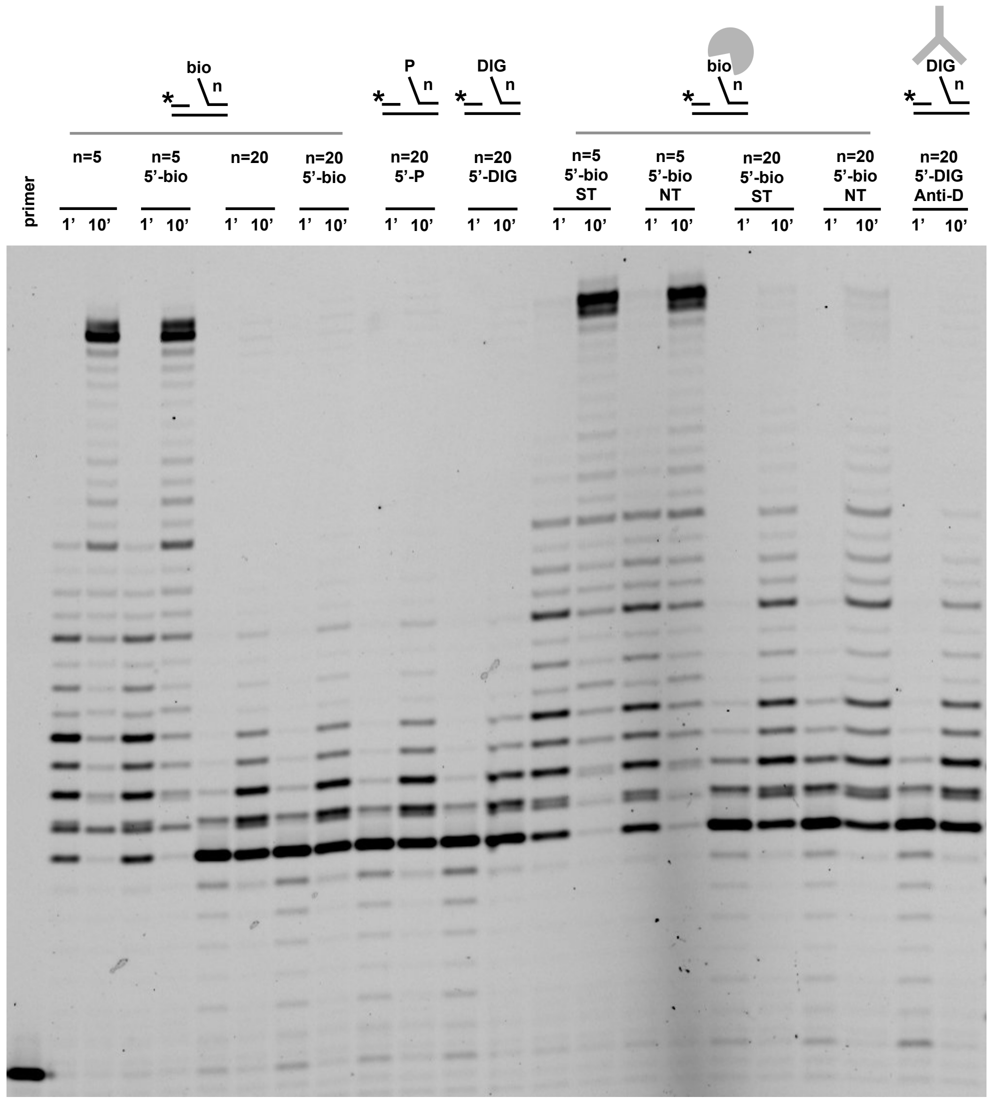

Figure S6. Strand displacement activity of Pol δ^{DV} in Buffer TM (20mM NaCl, 30 °C) using a T_1 gap substrate with the indicated length of the 5'-flap (n) without any modification or with 5'-biotin, 5'-digoxigenin. 5'-phosphate. For the biotinylated 5'-end the experiments where also performed in presence of excess of either streptavidin (ST) or neutravidin (NT). For the 5'-DIG on the flap the experiments where also in presence of an anti-DIG antibody. The 5 nt 5'flap is used as a control for the effect of each modification of the strand displacement activity.

ssDNA dT_{25} is not a good trap for Pol δ^{DV} and *in trans* inhibition does not originate exclusively from a ssDNA induced dissociation from the DNA substrate

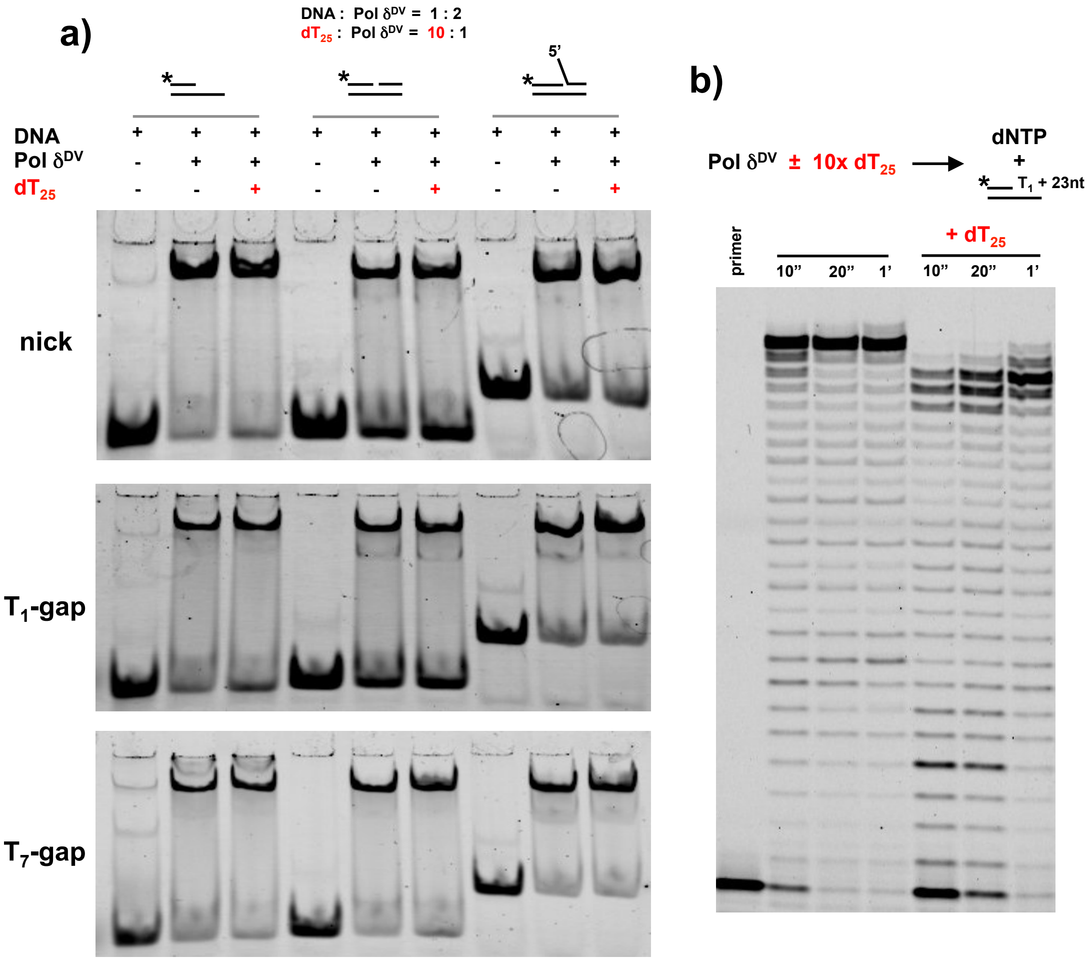

Figure S7. a) EMSAs of Pol δ complexes formed with the indicated DNA substrates (Cy3 labeled) in absence and presence of a 10-fold excess of dT_{25}. **b)** Primer extension activity in Buffer TM (20mM NaCl, 30 °C) of a reference, labeled primed template by Pol δ^{DV} after incubation in absence or presence of a 10-fold excess of dT_{25}.

SSB can substitute RPA in relieving the inhibitory effect of long 5'-flaps and the RPA effect is maintained with flaps of opposite orientation.

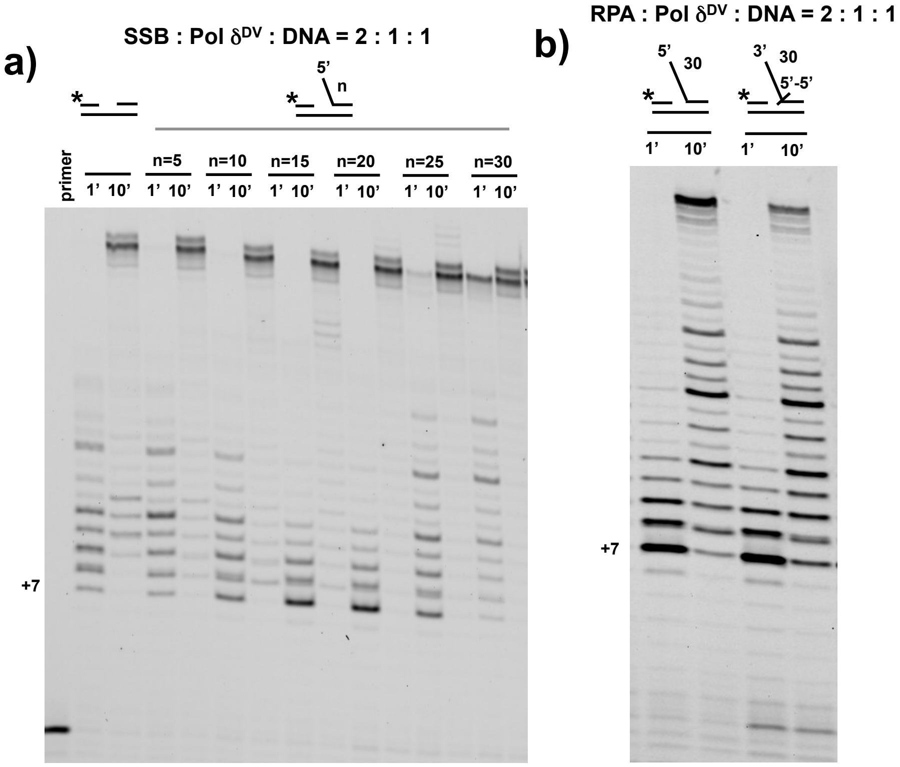

Figure S8. a) Effect of a SSB on the DNA primer extension and strand displacement activity of Pol δ^{DV} in Buffer TM (20mM NaCl, 30 °C) as function of the length (n) of the 5'-flap in the strand to be displaced using a T_7 gap substrate. **b)** Strand displacement activity of Pol δ^{DV} in Buffer TM (20mM NaCl, 30 °C) using a T_7 gap substrate containing either a 5'-flap or a 3'-flap of 30 nt.

Strand displacement synthesis by Pol δ can be monitored with a Cy3-Cy5 FRET pair.

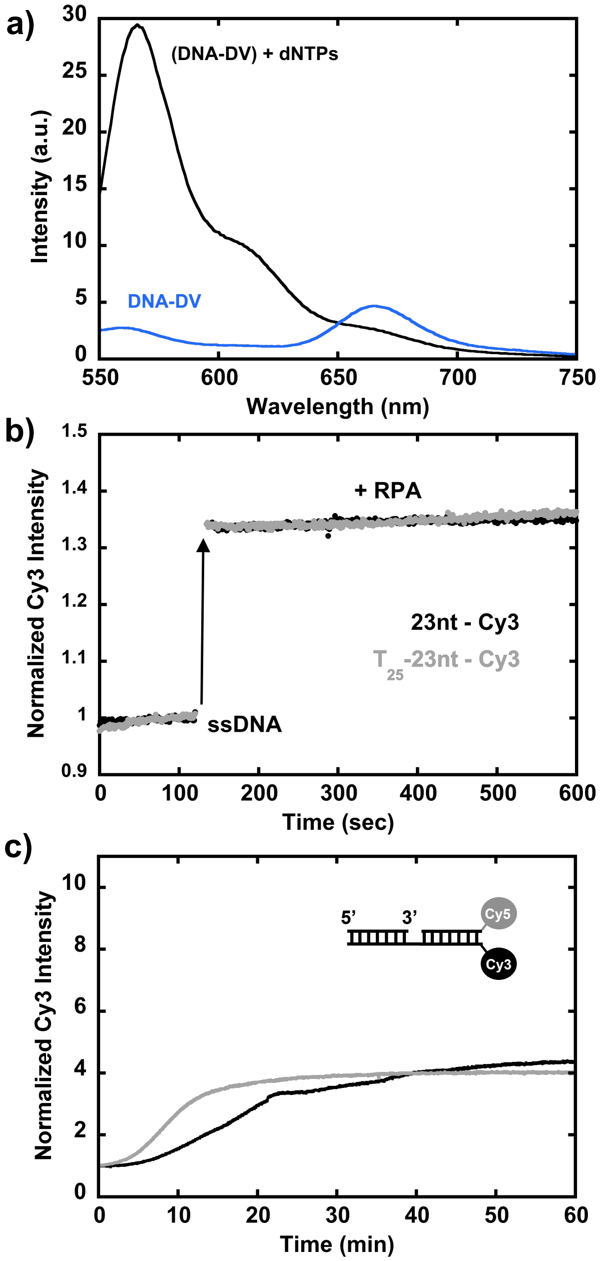

Figure S9. a) Fluorescence emission spectra (excitation 520 nm) before (grey) and after (black) adding dNTP to Pol δ^{DV} bound to a T_1 gap substrate (without a 5'-flap) that has a Cy3-Cy5 pair at the end of the duplex to be displaced (see Figure 4c). **b)** RPA-induced change in the fluorescence intensity of Cy3-labeled ssDNAs corresponding to the displaced strands in the Pol δ substrates. **c)** Time courses of Cy3 intensity (excitation at 520 nm and emission at 565 nm) using the T_1 gap substrate (without a 5'-flap) where the Cy3 and Cy5 at the end of the duplex where switched on the strands. The black trace is for a Pol δ^{DV}-only experiment and it has a t_{lag} of 6.2 minutes and $t_{1/2}$ of 18.3 minutes. The grey trace is for an experiment where RPA was presence from the beginning of the reaction and it has a t_{lag} of 3.4 minutes and $t_{1/2}$ of 9.1 minutes.

Strand displacement activity of Pol δ^{DV} and the effect of RPA are strongly temperature dependent.

The RPA stimulation of Pol δ^{DV} strand displacement synthesis at 20 °C rather than 30 °C, where the activity of the polymerase is lower (Figure S7a, Pol δ^{DV}-only *vs.* Pol δ^{DV}+RPA). The grey trace in Figure S7b shows the time course of the same experiment performed with the FRET-based assay. When RPA is present in solution from the beginning of the reaction the signal is characterized by a long lag-phase followed by linear increase. Similar to what observed at 30 °C (see main text), the two assays show a different time response and they are not directly compared but used to provide complementary information. When RPA is omitted from the reaction (Figure S7b, black trace) the signal hardly changes for ~30 min after addition of dNTPs, suggesting that either full product is not being released from the enzyme or that it is such a small fraction that it does not contribute to the signal. However, when RPA is added after ~ 30 min, the signal is characterized by an initial burst followed by a linear increase. In order to understand whether this burst originates from RPA binding to a small fraction of full ssDNA product or to the intermediates with shorter 5'-flaps, we performed the same experiment with the gel-based assay (Figure S7a, +RPA). The reaction with Pol δ^{DV}-only was allowed to proceed for 30 min, RPA was added and the time course monitored. The distribution of extended intermediates is similar to the one observed when RPA is present from the beginning. If binding of RPA to these intermediates were the main reason for stimulation of strand displacement, it would have been expected that the intermediates would be cleared faster when they are preformed by the polymerase. This also suggests that the small burst observed in the FRET-based assay likely originates from binding of RPA to a small fraction of ssDNA product generated.

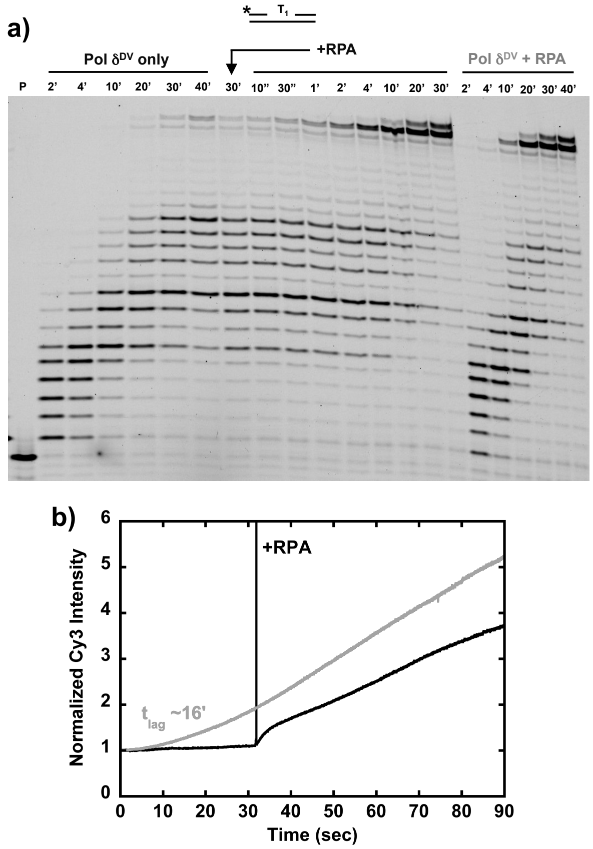

Figure S10. a) Strand displacement activity of Pol δ^{DV} in Buffer TM (20mM NaCl, 20 °C) using a T_1 gap substrate without a 5'-flap. For the Pol δ^{DV}-only experiments, RPA was added after 30 minutes. For Pol δ^{DV}+RPA, RPA was present from the beginning of the reaction. **b)** Experiments as in a) but using the fluorescence-based assay.

5'-flap and ssDNA "in trans" inhibition are maintained with a different version of exonuclease deficient DNA polymerase δ

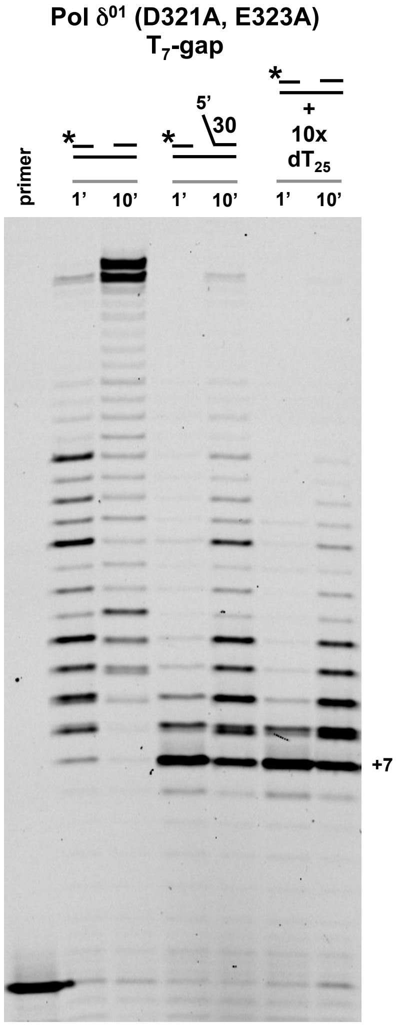

Figure S11. DNA primer extension and strand displacement activity of Pol δ^{01} (D321A, E323A) in Buffer TM (20mM NaCl, 30 °C) as a function of either a T_{30} 5'-flap or addition of a 10-fold excess of dT_{25} using a T_7 gap substrate.
